# Supplementary material for: Expression Patterns and Gonadotropin Regulation of the TGF-β II Receptor (Bmpr2) during Ovarian Development in the Ricefield Eel Monopterus albus
Source: Int J Mol Sci. 2022 Dec 5;23(23):15349. doi: 10.3390/ijms232315349 (PMC9739225; doi:10.3390/ijms232315349)
Supplement: Supplementary file 1 [file ijms-23-15349-s001.zip › ijms-2027853-supplementary.pdf]

Supplemental information

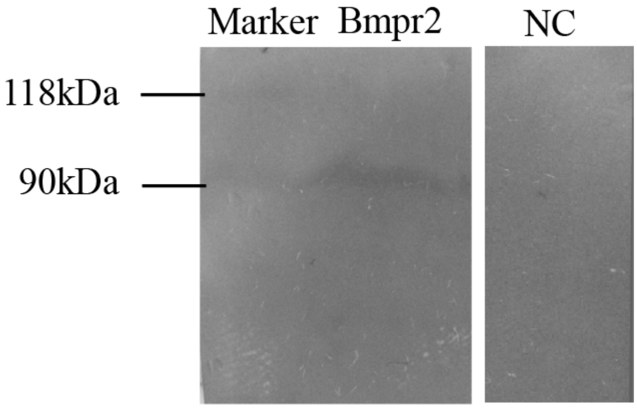

Figure S1. Bmpr2 immunoreactivity in the ovaries was evaluated using Western blot analysis. Marker, prestained protein Molecular Weight Marker. NC, negative control, the primary antibody was replaced with PBS in the negative control group.

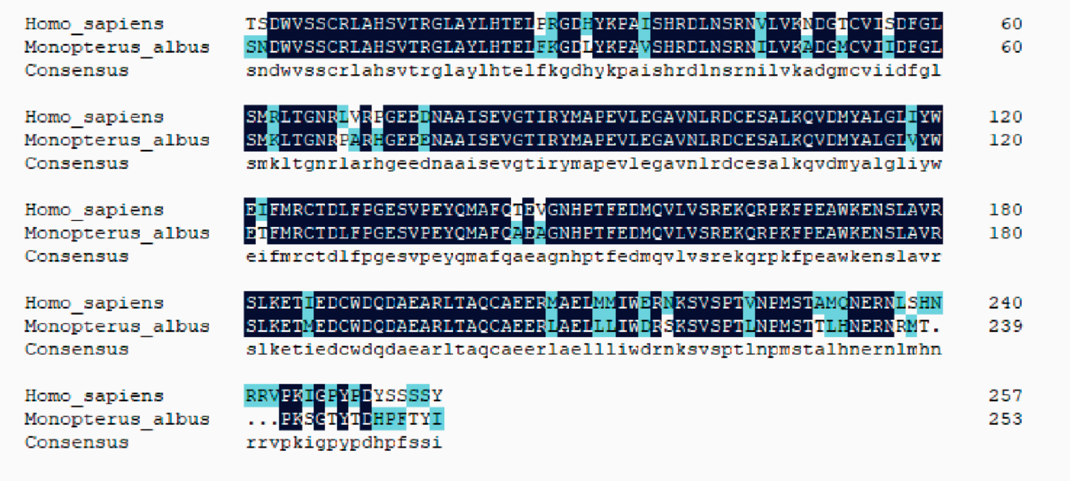

Figure S2. Amino acid sequence alignment of BMPR2 between *Homo sapiens* (XP_011509989.1, aa295-552) and *Monopterus albus*. Bmpr2. Conserved amino acid sequence were marked with different colors. Completely conserved amino acid sequences are represented in black color. The conservatism of amino acid sequence is greater than or equal to 50%, which are indicated in blue color.
